# Detection of SARS-CoV-2 Specific Antibodies in Saliva Samples

**DOI:** 10.3389/fimmu.2022.880154

**Published:** 2022-07-08

**Authors:** Siyang Yu, Peiyan Zhang, Mingfeng Liao, Juanjuan Zhang, Suisui Luo, Jinglei Zhai, Yaxi Zhang, Jingyan Lin, Jing Yuan, Zheng Zhang, Fuxiang Wang, Lanlan Wei

**Affiliations:** ^1^ Institute of Hepatology, National Clinical Research Center for Infectious Disease, The Third People’s Hospital of Shenzhen, The Second Hospital Affiliated to Southern University of Science and Technology, Shenzhen, China; ^2^ Clinical Laboratory, The Seventh Affiliated Hospital, Sun Yat-sen University, Shenzhen, China; ^3^ Department of Infectious Diseases, The Third People’s Hospital of Shenzhen, The Second Hospital Affiliated to Southern University of Science and Technology, Shenzhen, China; ^4^ School of Medicine, Southern University of Science and Technology, Shenzhen, China; ^5^ Shenzhen Research Center for Communicable Disease Diagnosis and Treatment of Chinese Academy of Medical Science, Shenzhen, China

**Keywords:** COVID-19, SARS-CoV-2, immunoglobulins, saliva IgA, diagnose

## Abstract

Molecular assays on nasopharyngeal swabs act as a confirmatory test in coronavirus disease (COVID-19) diagnosis. However, the technical requirements of nasopharyngeal sampling and molecular assays limit the testing capabilities. Recent studies suggest the use of saliva for the COVID-19 diagnostic test. In this study, 44 patients diagnosed with COVID-19 in The Third People’s Hospital of Shenzhen were enrolled. Saliva and serum specimens were obtained at different time points and the immunoglobulins against SARS-CoV-2 were measured. The results showed that saliva IgA presented a higher COI value than IgG and IgM. In matched saliva and serum samples, all saliva samples presented lower IgG levels than serum samples, and only one saliva sample presented a higher IgM level. The conversion rates of saliva IgA and the detection of viral nucleic acids were analyzed in the first and second weeks after hospitalization. The positive rates increased when combining saliva IgA and viral nucleic acid detection. In conclusion, our results provide evidence that saliva IgA could serve as a useful index for the early diagnosis of COVID-19.

## Introduction

The pneumonia outbreak in Wuhan, China in December 2019 was caused by the Severe Acute Respiratory Syndrome Coronavirus 2 (SARS-CoV-2). Currently, the coronavirus disease (COVID-19) pandemic is developing rapidly into a dramatically devastating public health crisis. By April 2021, reported cases of COVID-19 had exceeded 147 million worldwide, with at least 3,144,381 deaths. Molecular assays on nasopharyngeal swabs are confirmatory tests for COVID-19 diagnosis (1). Despite massive efforts, the positive rate of RNA detection for SARS-CoV-2 was 63% in nasopharyngeal swabs and only 32% in pharyngeal swabs (2). Serological assays play an important role in the clinical diagnosis of COVID-19. IgM and IgG-based assays are the gold standard for serological diagnosis in COVID-19 (3). SARS-CoV-2 S1 and N antigens have been detected in the serum of SARS-CoV-2 infected patients, which help detect active infection and monitor disease progression in COVID-19 patients (4).

Currently, nasopharyngeal swabs are the main recommended upper respiratory tract specimen types for the COVID-19 test, whereas the use of saliva for the diagnosis of the disease has recently been suggested (5, 6). Saliva specimens could be obtained conveniently. The collection of saliva is non-invasive and greatly minimizes the exposure of healthcare workers to COVID-19 (7). The detection of SARS-CoV-2 salivary antibodies could serve as a non-invasive alternative to serological tests (8). Saliva is secreted by salivary glands, which is characteristic of abundant IgA. Usually, salivary IgG and IgM concentrations are much lower than those in the serum (9). It has been hypothesized that both salivary IgG and IgM are derived from blood, whereas IgA is mainly produced by the salivary glands (10).

A recent study reported that salivary IgA was associated with the presence of pneumonia but unassociated with serum immunoglobulins (11). These results suggest that salivary IgA is independent of serum immunoglobulins. In this study, we measured saliva and serum specimens from 44 COVID-19 patients and 24 negative control patients. The associations between saliva and serum immunoglobulins were analyzed and the potential of saliva IgA in COVID-19 diagnosis was assessed.

## Materials and Methods

### Patients

A total of 44 patients diagnosed with COVID-19 based on the World Health Organization’s interim guidance, from 1 August to 1 September 2020, at The Third People’s Hospital of Shenzhen were enrolled in this study. A total of 24 negative-control patients with no SARS-CoV-2 infection were selected randomly from inpatient departments. The study was approved by the Ethics Committee of The Third People’s Hospital of Shenzhen. Written informed consent was obtained from all participants enrolled in the study.

### Immunoglobulin Measurement

A total 180 of saliva specimens and 181 peripheral blood specimens were obtained from COVID-19 patients with RT-PCR confirmed prior SARS-CoV-2 infection, at different time points during hospitalization. Saliva specimens and peripheral blood specimens were also obtained from negative-control patients. The serum specimens were obtained from the supernatant of centrifuged peripheral blood at 3,500 rpm for 5 min. The saliva specimens were centrifuged and the supernatants were collected for immunoglobulin detection. All specimens were inactivated at 56 °C for 30 min. Immunoglobulins against SARS-CoV-2 surface spike protein receptor-binding domain (RBD) were measured by chemiluminescence kit (IgA, IgG, and IgM, Beijing Wantai Biotech, China) according to the instructions of the manufacturer. The relative fluorescence of the sample to control (COI) was used to estimate the result, with COI ≥1 as positive and <1 as negative.

### Real-Time PCR

Over 240 swab samples were obtained from the upper respiratory tracts of participants, and SARS-CoV-2 was detected by RT-PCR assay as reported previously. Briefly, the nucleocapsid protein and open reading frame 1ab were amplified and examined with two pairs of primers. Each sample was detected in triplicate with positive and negative controls. The diagnostic criteria were based on the recommendations by the National Center for Disease Control and Prevention of China.

### Statistical Analysis

Statistical analysis was performed using SPSS software version 22.0. A Student’s t-test was used to compare the difference between different antibodies in saliva. A paired t-test was used to analyze the difference in antibody COI between serum and saliva.

## Results

**Figure 1:**
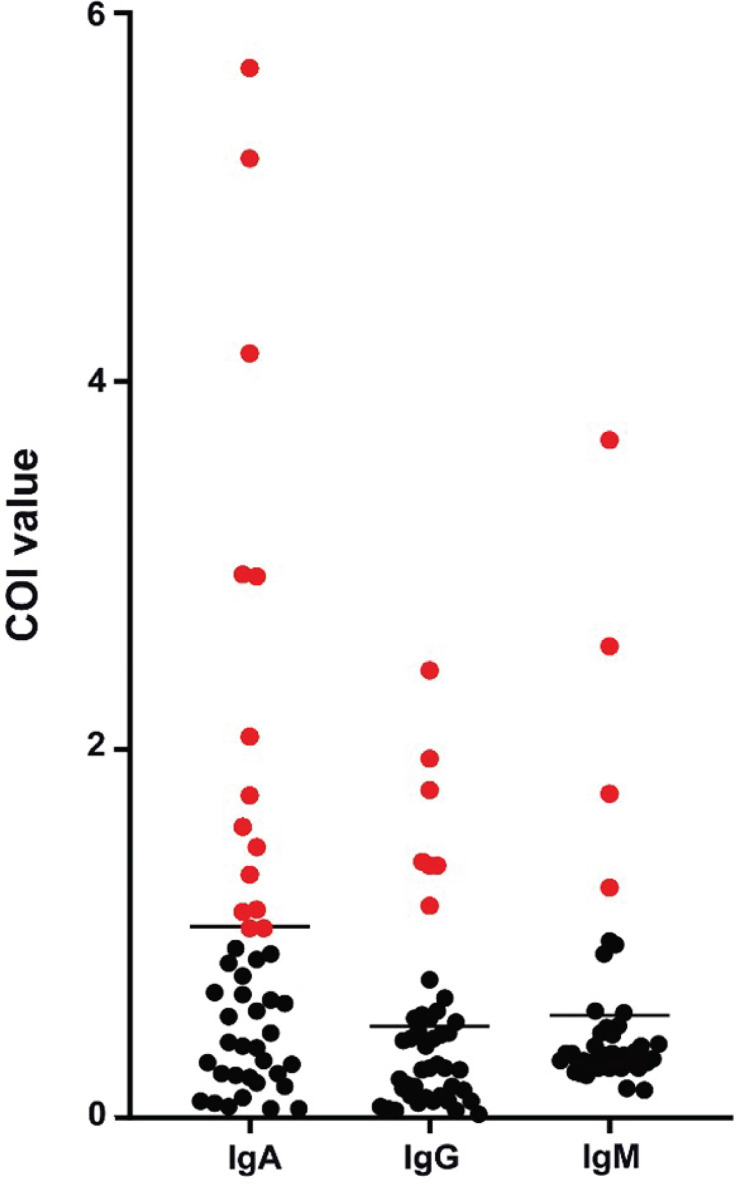
Peak levels of saliva immunoglobulins in COVID-19 patients. Each point presented the highest measured COI value of immunoglobulin in saliva of each patient. Positive results were colored in red.

**Figure 2:**
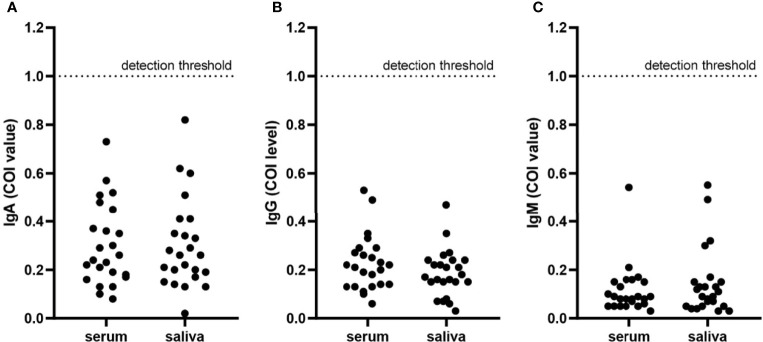
Immunoglobulins in serum and saliva specimens from negative-control patients. Each point presented the COI value of IgA **(A)**, IgG **(B)**, and IgM **(C)** in serum or saliva specimens of each negative-control patient. The detection threshold was marked in each figure at COI = 1.

Patients diagnosed with COVID-19 from 1 August to 1 September 2020 at The Third People’s Hospital of Shenzhen were enrolled in this study (n = 44). The characteristics, including age, gender, and disease severity, are listed in **Table 1**. Most patients were male and asymptomatic. The average age of the patients was 43 years (a range of 22–62 years). Saliva and serum from patients were collected and the levels of IgA, IgG, and IgM were measured. The highest COI value of each patient was used to represent the immunoglobulin level in their saliva or serum. As shown in **Figure 1** and **Table 2**, 14 patients presented with positive for IgA in saliva, whereas 7 and 4 patients presented with positive for IgG and IgM, respectively. Moreover, IgA presented a higher COI value than IgG and IgM in saliva (*p* = 0.0128 and *p* = 0.0297, respectively). IgA, IgG, or IgM in saliva and serum specimens were all negative for 24 negative-control patients (**Figure 2**).

**Table 1:** Characteristics of enrolled patients.

	Male (n = 40)	Female (n = 4)
Age [median, (range)]	43 (29–59)	53 (37–62)
Disease severity [n, (%)]		
asymptomatic	39 (97.5%)	2 (50.0%)
moderate	1 (2.5%)	1 (25.0%)
severe	0 (0.0%)	1 (25.0%)
Sampling time [median, (range)]	2 (1–13)	2 (1–6)
Complications [n, (%)]		
hypertension	5 (12.5%)	0 (0.0%)
hyperlipidemia	2 (5.0%)	0 (0.0%)
diabetes	1 (2.5%)	0 (0.0%)
tumor	1 (2.5%)	0 (0.0%)
intestinal diseases	2 (5.0%)	0 (0.0%)

**Table 2:** Positive rate of immunoglobulins in saliva.

Immunoglobulin	Positive (+)	Negative (−)	total	Positive rate (%)
IgA	14	30	44	31.82
IgG	7	37	44	15.91
IgM	4	40	44	9.09

Saliva and serum which were collected on the same day or on two consecutive days were analyzed as matched samples (n = 15) **(** **Table 3** **)**. A total of 5 saliva specimens presented higher IgA levels than matched serum. Generally, IgA in saliva specimens showed roughly the same level as in serum (saliva, 11 positive vs. 4 negative; serum, 10 positive vs. 5 negative). IgG and IgM levels in saliva specimens were lower than those in serum (*p* <0.0001 and *p* = 0.0444, respectively). All saliva presented lower IgG levels than serum (saliva, 5 positive vs. 10 negative; serum, 15 positive vs. 0 negative), and only one saliva specimen presented a higher IgM level (saliva, 3 positive vs. 12 negative; serum, 5 positive vs. 10 negative). No clear correlation was observed among the IgA, IgG, and IgM-positive samples.

**Table 3:** The collection time and results of paired serum and saliva specimens.

Serum							Saliva						
Collection time (days)	IgA		IgG		IgM		Collection time (days)	IgA		IgG		IgM	
2	0.98	(−)	8.08	(+)	4.52	(+)	3	0.35	(−)	0.43	(−)	1.76	(+)
2	2.14	(+)	12.6	(+)	5.17	(+)	3	1.03	(+)	0.46	(−)	0.27	(−)
4	3.93	(+)	7.55	(+)	0.2	(−)	3	1.32	(+)	0.65	(−)	0.17	(−)
5	1.88	(+)	20.68	(+)	6.82	(+)	4	1.75	(+)	1.78	(+)	2.56	(+)
5	0.37	(−)	8.53	(+)	0.85	(−)	4	1.12	(+)	0.54	(−)	0.44	(−)
7	1.15	(+)	2.24	(+)	4.8	(+)	6	1.13	(+)	0.04	(−)	0.12	(−)
8	4.85	(+)	18.82	(+)	3.43	(+)	7	0.14	(−)	1.37	(+)	0.21	(−)
8	4.5	(+)	5.64	(+)	0.16	(−)	8	4.7	(+)	0.3	(−)	0.04	(−)
9	1.21	(+)	17.74	(+)	0.24	(−)	10	1.47	(+)	1.95	(+)	0.29	(−)
11	1.36	(+)	10.3	(+)	0.08	(−)	10	5.21	(+)	0.04	(−)	0.04	(−)
11	0.66	(−)	3.49	(+)	0.45	(−)	12	0.09	(−)	1.37	(+)	0.22	(−)
12	2.42	(+)	12.36	(+)	0.28	(−)	12	0.41	(−)	2.43	(+)	0.13	(−)
13	0.71	(−)	12.98	(+)	0.41	(−)	12	1.23	(+)	0.19	(−)	0.25	(−)
14	6.06	(+)	5.66	(+)	0.26	(−)	13	4.15	(+)	0.54	(−)	2.68	(+)
19	0.99	(−)	3.8	(+)	0.12	(−)	20	1.03	(+)	0.16	(−)	0.12	(−)

The first column of each type of immunoglobulins was COI value and the second column was the qualitative result. (+) means positive and (−) means negative.

To investigate whether the test of saliva IgA could improve the diagnostic power of COVID-19 patients, the conversion rates of saliva IgA and the detection of viral nucleic acids were analyzed in the first and second weeks after hospitalization (n = 39) (**Table 4**). While the patients were hospitalized with positive nucleic acid results at the beginning, the positive rate was as low as 35.90% in the first week and 12.82% in the second week. The positive rates increased with saliva IgA.

**Table 4:** Positive detection rate of SARS-CoV-2 nucleic acid and saliva IgA at different time periods.

Time (days)	RNA		saliva IgA		RNA or saliva IgA	
	n	positive rate (%)	n	positive rate (%)	n	positive rate (%)
1–7	14	35.90	6	15.38	19	48.72
8–14	5	12.82	3	7.69	8	20.51
1–14	15	38.46	8	20.51	20	51.28

## Discussion

This study investigated the use of saliva for detecting SARS-CoV-2 specific antibodies from COVID-19 patients. This study was conducted at The Third People’s Hospital of Shenzhen in September 2020, so most patients enrolled were in the recovery phase of the disease. This may explain why the percentage positive rate of SARS-CoV-2 nucleic acid in our inpatient series was low.

Saliva has been used over decades for evaluating human health with several advantages in that it is a noninvasive, painless, safe, and convenient specimen (12, 13). Pisanic et al. tested SARS-CoV-2-specific IgA, IgG, and IgM in saliva specimens with a considerable detection rate (8). In an Australian family case, saliva antibodies were detected in all family members (14). In our study, despite the low detection rate, IgA, IgG, and IgM were all detectable in saliva specimens.

Secretory IgA is a principal component of mucosal immunity and can be easily measured in saliva (15). IgA has been proved to be the dominant antibody in early SARS-CoV-2-specific humoral response (16). Salivary IgA antibody responses were reported to be particularly prevalent in younger individuals with mild SARS-CoV-2 infection (17). Similarly, we found that the level and detection rate of IgA in saliva were higher than IgG and IgM. The analysis of saliva and serum SARS-CoV-2-specific antibodies showed that IgA, IgG, and IgM levels in matched saliva and serum samples were all significantly correlated (8). However, IgA levels in the saliva exhibited the poorest correlation with IgA levels in the serum (18). In our study, levels of IgG and IgM in saliva were lower than in serum, and we found no clear correlation between IgA levels in paired saliva and serum samples.

Recently, saliva has been proposed as a suitable specimen for the diagnosis of COVID-19, and the collection method would reduce the exposure risk of frontline health workers (19). SARS-CoV-2 RNA could remain detectable in saliva over a 1-week period, but the test is unstable and vulnerable (20, 21). Neutralizing IgA was reported to remain detectable in saliva for a longer time (days 49 to 73 post symptoms) than in serum (16). Our results showed that testing antibodies against SARS-CoV-2 was sensitive in saliva samples, providing an easy, noninvasive option for detecting viral infection. The combination of an antibody test on saliva and traditional molecular assays on nasopharyngeal swabs could provide the diagnostic ability. Additionally, the increased salivary IgA has been proposed as a biomarker to identify patients at an elevated risk of clinical deterioration in COVID-19 (15). All the evidence suggests that IgA in saliva could play an important role in COVID-19 diagnosis.

However, we should note that SARS-CoV-2 antibody is present in various clinical specimens such as serum, plasma, nasopharynx, oropharynx, nose, ocular fluid, sputum, bronchoalveolar lavage, urine, and stool, in addition to saliva. A recent review summarized the relative detection rate of SARS-CoV-2 antibodies in different specimens in detail, and the authors concluded that the infectious potential of these specimens mainly depended on the time of specimen collection and the presence of live replicating viral particles (22). Greater detection sensitivity and consistency have been achieved in saliva samples during infection than in nasopharyngeal samples (2). A meta-analysis comparing paired saliva and nasopharyngeal samples in confirmed cases showed a positive detection rate of 88% for saliva samples and 94% for nasopharyngeal swabs, without a significant difference (23). Another meta-analysis showed an overall diagnostic accuracy of 92.1% with sensitivity of 83.9% and specificity of 96.4% for saliva samples compared with nasopharyngeal/oropharyngeal samples in confirmed cases (24). However, another meta-analysis reported that the sensitivity of saliva samples was 3.4% lower than that of nasopharyngeal swabs (25). Further studies are necessary to compare the efficacy of detection of SARS-CoV-2 antibodies in saliva samples with other samples of body fluids.

This study has several limitations. First, the concentration of antibodies in human saliva is significantly lower than that in the blood or serum. Therefore, assays with exquisite analytical sensitivity to detect high signals over background noise are required (26). Second, our sample set was not large enough, especially lacking the samples at early time points. In addition, antibody levels in patients with asymptomatic infections are always lower than in patients with symptomatic infections. Future studies could improve the robustness by including a larger sample size at all time points.

## Data Availability Statement

The original contributions presented in the study are included in the article/supplementary material. Further inquiries can be directed to the corresponding author.

## Ethics Statement

The studies involving human participants were reviewed and approved by the Third Hospital of Shenzhen. The patients/participants provided their written informed consent to participate in this study.

## Author Contributions

SY, PZ, ML, JZ, SL, JZ, YZ, JL, and JY collected and analyzed the samples. ZZ, FW, and LW designed and supervised the study. All authors listed have made a substantial, direct, and intellectual contribution to the work and approved it for publication.

## Funding

This research was funded by the Shenzhen Fund for Guangdong Provincial High-level Clinical Key Specialties (Nos. SZGSP010 and SZGSP011).

## Conflict of Interest

The authors declare that the research was conducted in the absence of any commercial or financial relationships that could be construed as a potential conflict of interest.

## Publisher’s Note

All claims expressed in this article are solely those of the authors and do not necessarily represent those of their affiliated organizations, or those of the publisher, the editors and the reviewers. Any product that may be evaluated in this article, or claim that may be made by its manufacturer, is not guaranteed or endorsed by the publisher.
